# Non-Uniformity Correction of Spatial Object Images Using Multi-Scale Residual Cycle Network (CycleMRSNet)

**DOI:** 10.3390/s25051389

**Published:** 2025-02-25

**Authors:** Chunfeng Jiang, Zhengwei Li, Yubo Wang, Tao Chen

**Affiliations:** 1 Changchun Institute of Optics, Fine Mechanics and Physics, Chinese Academy of Sciences, Changchun 130033, China; jiangcf6512@163.com (C.J.);; 2 University of Chinese Academy of Sciences, Beijing 100049, China; chent@ciomp.ac.cn

**Keywords:** ground-based telescopes, non-uniform image, CycleMRSNet, multi-scale attention

## Abstract

Ground-based telescopes often encounter challenges such as stray light and vignetting when capturing space objects, leading to non-uniform image backgrounds. This not only weakens the signal-to-noise ratio for target tracking but also reduces the accuracy of recognition systems. To address this challenge, We have proposed a novel network architecture called CycleMRSNet, which is based on the CycleGAN framework and incorporates a multi-scale attention mechanism to enhance image processing capabilities. Specifically, we have introduced a multi-scale feature extraction module (MSFEM) at the front end of the generator and embedded an efficient multi-scale attention residual block (EMA-residual block) within the Resnet backbone network. This design improves the efficiency of feature extraction and increases the focus on multi-scale information in high-dimensional feature maps, enabling the network to more comprehensively understand and concentrate on key areas within images, thereby capably correcting non-uniform backgrounds. To evaluate the performance of CycleMRSNet, we trained the model using a small-scale dataset and conducted corrections on simulated and real images within the test set. Experimental results showed that our model achieved scores of PSNR 32.7923, SSIM 0.9814, and FID 1.9212 in the test set, outperforming other methods. These metrics suggest that our approach significantly improves the correction of non-uniform backgrounds and enhances the robustness of the system.

## 1. Introduction

Ground-based optical telescopes play a crucial role in the tracking and recognition of space objects. However, as the aperture of these telescopes increases, the vignetting effect within the optical system becomes more pronounced. This effect weakens the energy of targets at the edges of images, which is detrimental to target recognition. Additionally, stray light can also degrade the overall image quality. Both the vignetting and stray light contribute to the non-uniform background of the images. This can reduce the final image quality and signal-to-noise ratio, causing some targets to be submerged and indistinguishable. Therefore, it is necessary to perform non-uniform background correction on images of space objects to meet the critical tasks of detection, tracking, and recognition.

Image vignetting refers to the phenomenon where the brightness of an image gradually decreases from the optical center towards the edges [1]. This effect is primarily determined by the parameters of the optical system and the lens. In visible light images of space objects, the main source of stray light is non-target sources outside the field of view, such as observations made before dawn or after dusk, or when the field of view is near the Moon. The combination of vignetting and stray light results in a non-uniform background in the images, which severely interferes with subsequent tasks of target detection and recognition. Thus, addressing these issues through effective correction is vital.

In the field of image non-uniformity correction, the primary methods include flat-field correction and image-processing techniques. Flat-field correction [2] involves using a standard flat-field image captured under uniform illumination to correct the vignetting effect in actual images. However, in large-field-of-view (LFOV) detection systems, obtaining a uniform light source that can illuminate the entire field of view is challenging. Additionally, the complex optical design of LFOV systems often leads to significant variations in vignetting effects across different positions, making flat-field correction less effective in such scenarios.

Image processing methods for vignetting correction can primarily be categorized into single-frame-based approaches and multi-frame-based approaches. Multi-frame methods involve extracting vignetting information from multiple images of the same scene and shooting conditions to construct a vignetting model. This model is then used, in conjunction with specific algorithms, to compensate for non-uniformities within the images, thereby achieving vignetting correction. Harris et al. [3] proposed a constant statistics method that requires a long video sequence to ensure the convergence of non-uniformity correction coefficients. Meanwhile, Torres S.N. et al. [4] introduced a Kalman filtering approach. In this method, the gain and bias of each detector response element are treated as random variables generated by a Gaussian–Markov process. A Kalman filter based on the Gaussian–Markov model is designed to estimate and iteratively update the gain and bias of the response elements.

In recent years, with the rapid development of deep learning, an increasing number of deep learning algorithms have been widely applied in the field of optics and optical computing [5,6,7,8]. For instance, these applications include super-resolution imaging [9,10,11,12,13], image segmentation, optical interferometry techniques, wavefront aberration correction [14,15,16], and remote sensing [17,18,19,20,21,22]. Utilizing deep learning for processing non-uniform backgrounds and noise removal in images has become increasingly popular. Kuang et al. [23] proposed a single-frame infrared image non-uniformity correction algorithm based on deep convolutional neural networks. He et al. [24] utilized residual networks to create a non-uniformity residual image, which is then subtracted from the original image to obtain the corrected image. Jian et al. [25] combined bilateral filtering with residual networks, which performs well when non-uniformities exhibit high-frequency characteristics. Fang et al. [26] employed a U-net network for correcting non-uniform noise in images.

Zhang et al. [27] introduced a GAN network with a difference-aware alternative implementation mechanism, which encourages the network to focus on areas where correction might be less effective and simultaneously improves depth estimation performance in hard-to-recover regions of noisy images, thereby collaboratively aiding in image recovery. Sun et al. [28] proposed a Bidirectional Feature Fusion Generative Adversarial Network (BFF-GAN) that eliminates blocky artifacts in medical image reconstruction through global and local feature fusion and a patch-based attention mechanism, learning connections between patches. J. Kim et al. [29] developed a novel attention mechanism module for unsupervised image-to-image translation. This attention module guides the model to distinguish more critical areas between the source and target domains based on attention maps obtained from an auxiliary classifier. Guo et al. [30] presented an enhanced conditional GAN for correcting non-uniform backgrounds in optical images. This method effectively addresses non-uniform background issues caused by vignetting and stray light to some extent but it is a supervised learning approach requiring paired data for training and large datasets for manual annotation, leading to suboptimal robustness and computational resource requirements.

In recent years, Vision Transformers (ViTs) [31] and diffusion models have shown significant potential in the field of image processing. However, their applicability to the specific task of optical image non-uniformity correction still needs to be verified based on scenario requirements and extensive datasets. This is especially true for tasks that are sensitive to local details, such as the fine correction of vignetting area edges or presenting multi-scale changes in a vignetting background, which may require additional design improvements, like the window attention mechanism of the Swin Transformer [32]. Although such improvements can enhance local modeling capabilities, they may also impose constraints in scenarios with limited computational resources. The compatibility of these methods with unsupervised training approaches and the research problem at hand requires further validation. Based on the above analysis, this study chooses to use Generative Adversarial Networks (GANs), particularly adopting CycleGAN [33] as its foundational architecture. The core advantages of CycleGAN are reflected in two aspects: first, its cycle consistency loss function allows the model to learn the mapping relationship between two domains without paired datasets, which is highly compatible with the actual scenario of this task where it is difficult to obtain strictly paired non-uniform image datasets; second, compared to other GAN variants like StarGANv2 [34], CUT [35], and U-GAT-IT [29], the structure of CycleGAN is more straightforward, making it more flexible for modifications tailored to this task. However, the classic CycleGAN tends to under-correct or overly smooth images under complex background interference, especially in scenes co-interfered by vignetting and stray light.

To address these limitations, we propose a new network model called the Multi-Scale Residual Cycle Network (CycleMRSNet). This network is capable of handling various intensities of non-uniform backgrounds in optical spatial target images across multiple scales. It combines the advantages of multi-scale attention mechanisms and cross-space learning, operates as an unsupervised learning model, and achieves better correction effects using only a small amount of unpaired data.

The remainder of this paper is organized as follows: In Section 2, we introduce the architecture of our CycleMRSNet model, detailing the working principles of the multi-scale feature extraction module (MSFEM) and the implementation of the efficient multi-scale attention residual block (EMA-residual block). Additionally, we describe the methodology used to create our simulation dataset. Section 3 outlines our experimental procedures and results. This section includes the metrics employed for evaluation, the ablation studies conducted to validate the effectiveness of our model, and comparisons of correction outcomes on both simulated test sets and real images with other methods, thereby demonstrating the feasibility and efficacy of CycleMRSNet. In Section 4, we discuss the results obtained using CycleMRSNet and summarize the work and contributions of this paper.

## 2. Methods

### 2.1. Network Architecture

**Figure 1:**
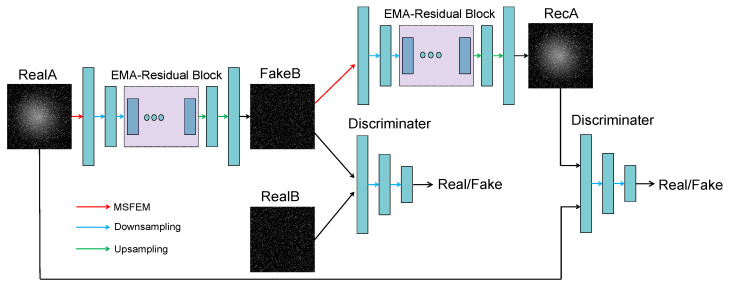
The overall structure of the network. The MSFEM is the multi-scale feature extraction module in the initial layer of the network, while the EMA-residual block is a feature extraction residual block that incorporates efficient multi-scale attention [36]. These details will be covered in the next subsection.

Our overall network framework design adheres to the fundamental principles and architecture of CycleGANs (Cycle-Consistent Adversarial Networks) [33], comprising a pair of symmetric generators and discriminators. As illustrated in Figure 1, this figure depicts the workflow of one path within the generator and discriminator system. We define images with non-uniform backgrounds as belonging to domain X, while clean images are designated as domain Y. Consequently, the problem of non-uniform background correction can be framed as a mutual transformation generation task between these two image domains.

In our network’s single pathway, real images RealA from domain X are mapped to FakeB by generator G and sent along with real images RealB from domain Y to the discriminator for authenticity evaluation. This part of the loss function is referred to as the adversarial loss, and we express the objective as follows:

(1) $$\begin{array}{cc} L_{\mathrm{GAN}}\left(G,D_{Y},X,Y\right) & =E_{y∼p_{\mathrm{data}\;}\left(y\right)}\left[\log D_{Y}\left(y\right)\right]\\ & +E_{x∼p_{\mathrm{data}\;}\left(x\right)}\left[\log\left(1-D_{Y}\left(G\left(x\right)\right)\right]\right. \end{array}$$

Subsequently, FakeB is further transformed back into an image RecA in domain X via generator F and is re-evaluated alongside RealA using the discriminator to assess their authenticity once more. This component of the loss function is known as the cycle consistency loss, detailed in:

(2) $$\begin{array}{cc} L_{\mathrm{cyc}\;}\left(G,F\right) & =E_{x∼p_{\mathrm{data}\;}\left(x\right)}\left[{∥F\left(G\left(x\right)\right)-x∥}_{1}\right]\\ \; & +E_{y∼p_{\mathrm{data}\;}\left(y\right)}\left[{∥G\left(F\left(y\right)\right)-y∥}_{1}\right] \end{array}$$

Additionally, we introduce an extra term, identity loss, which measures the distance between the original image RealB in domain Y and its transformed version G(B) produced by generator G. The goal is to ensure that even when images from domain Y are fed into generator G, they maintain the stylistic characteristics of domain Y. The specific expression for this loss is provided in:

(3) $$\begin{array}{cc} L_{\mathrm{Identity}\;}\left(G,F\right) & =E_{y∼p_{\mathrm{data}\;}\left(y\right)}\left[{∥G\left(y\right)-y∥}_{1}\right]\\ \; & +E_{x∼p_{\mathrm{data}\;}\left(x\right)}\left[{∥F\left(x\right)-x∥}_{1}\right] \end{array}$$

So, the overall loss function of our network is the weighted sum of the previous loss functions, expressed as:

(4) $$\begin{array}{cc} L\left(G,F,D_{X},D_{Y}\right) & =L_{\mathrm{GAN}}\left(G,D_{Y},X,Y\right)\\ \; & +L_{\mathrm{GAN}}\left(F,D_{X},Y,X\right)\\ \; & +\lambda L_{\mathrm{cyc}}\left(G,F\right)\\ \; & +\lambda \cdot \lambda_{\mathrm{idt}\;}L_{\mathrm{Identity}\;}\left(G,F\right) \end{array}$$

where λ represents the weight coefficient of the cycle consistency loss and is set to 10 in our task, while the identity loss weight coefficient $\lambda_{\mathrm{idt}\;}$ is uniformly set to 0.5.

### 2.2. MSFEM (Multi-Scale Featrue Extraction Module)

**Figure 2:**
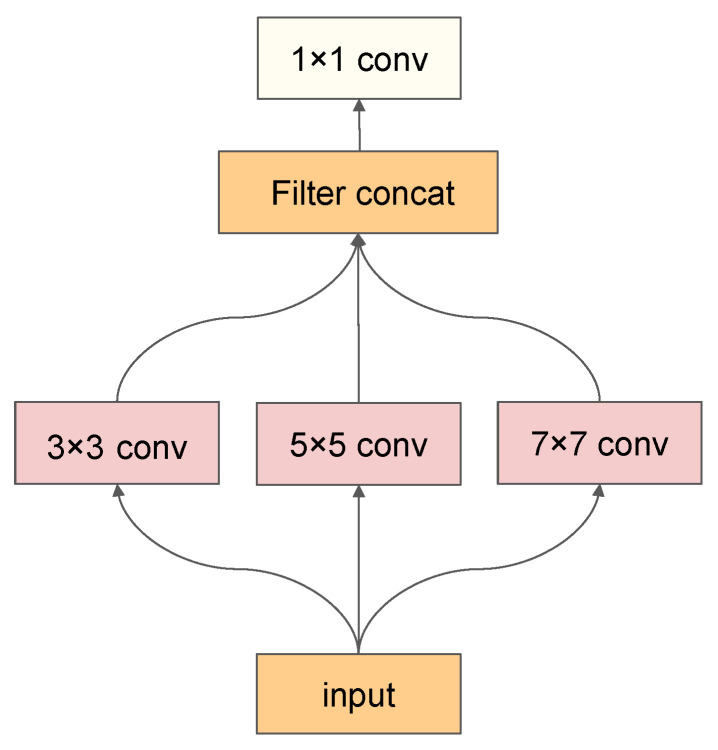
Multi-scale feature extraction module (MSFEM) architecture.

Inspired by the research of Wang et al. [37] and the core philosophy of the Inception [38] architecture, we propose an innovative multi-scale feature extraction module that integrates the advantages of large-kernel convolutions with the multi-scale feature fusion characteristics of the Inception model. Specifically, in the initial layer of the network, we replace the traditional 7 × 7 convolution operation with a module that is capable of multi-scale fusion. As shown in Figure 2, by employing three different sizes of convolution kernels—3 × 3, 5 × 5, and 7 × 7—we achieve feature capture across multiple scales of the input data, effectively integrating short-range and long-range dependencies. To preserve edge information, this module utilizes reflection padding, followed by pointwise convolutions on the feature channel dimension to facilitate feature integration and reduce computational load for subsequent processing. Each convolution operation is succeeded by an Instance Normalization (IN) layer and a ReLU activation function layer to enhance the non-linear representation capability of the model.

In scenarios where computational resources are limited, depthwise separable convolutions can be used as an alternative to large-kernel convolutions, significantly reducing computational complexity. Alternatively, multiple consecutive 3 × 3 convolution operations can serve as a substitute for large-kernel convolutions. However, the latter may necessitate adjustments to the training strategy to ensure that model performance is not compromised.

### 2.3. EMA-Residual Block

Before performing network downsampling and high-dimensional feature extraction, we utilize a MSFEM to capture initial image features. However, when dealing with high-dimensional features (i.e., a large number of channels) and large feature maps (often due to high-resolution input images), this approach can lead to a significant increase in parameters and computational costs. Therefore, there is an urgent need for a more efficient method that not only focuses on multi-scale information but also integrates attention across both the channel and spatial dimensions of high-dimensional feature maps.

In recent years, within the field of computer vision, channel and spatial attention mechanisms have been increasingly recognized as crucial for generating clearer, more detailed, and realistic images. Building on the design philosophy of Inception, which posits that different convolutional kernels can extract information at various scales from feature maps, a notable advancement was introduced by Ouyang et al., (2023) [36]. They proposed a novel, efficient multi-scale attention (EMA) mechanism based on Coordinate Attention (CA). This mechanism not only inherits the advantages of the Coordinate Attention mechanism found in the CA block but also adopts the multi-branch concept from Inception. Specifically, it introduces a bypass with a 3 × 3 convolution, to achieve information fusion across different scales.

The EMA mechanism divides the feature maps into groups and processes them through two parallel branches, maintaining the channel dimension. This allows for cross-spatial learning and enhances pixel-level attention in higher-level feature maps [39]. Finally, the outputs from multiple branches are combined via element-wise multiplication, with residual connections added to maintain training stability.

**Figure 3:**
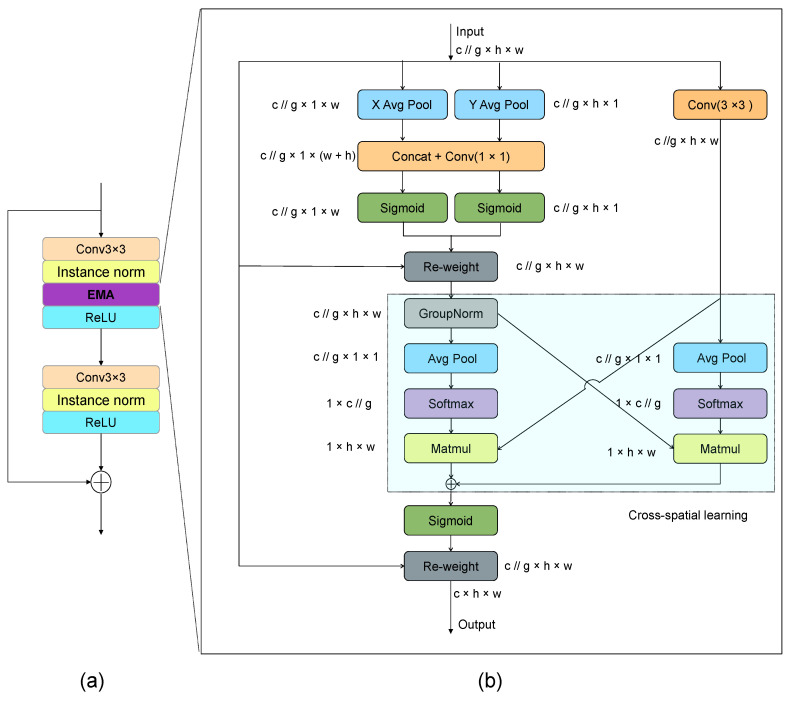
This is an EMA-residual block. (**a**) The EMA position in the residual block; (**b**) the archetecture of the EMA Module.

As shown in Figure 3. Our model takes multiple feature maps from each stage as input. Initially, these feature maps are divided into some groups and then processed through two parallel paths. In the first path, 1 × 1 convolutions are applied, followed by global average pooling along the H and W dimensions of the feature maps to extract weights in different directions, which are subsequently merged. This process draws inspiration from the CA (Coordinate Attention) mechanism. The second path involves 3 × 3 convolution operations, followed by global average pooling and the use of a softmax function to integrate the weights from this path. Ultimately, cross-spatial learning methods are employed to capture dependencies between all channels and cross-spatial information, achieving richer and more comprehensive information fusion. This parallel branch design enhances computational efficiency.

To further improve model performance, we have integrated an EMA module into the original residual block, specifically placing it after the normalization layer following the first 3×3 convolution operation. The resulting EMA-residual block, as illustrated in Figure 3a, maintains the high efficiency, lightweight nature, and strong generalization capabilities of the EMA module while effectively boosting the model’s expressive power.

### 2.4. Synthetic Datasets

In this task, we need to use simulated data to construct a dataset that is suitable for our algorithm. The dataset consists of two parts: one part comprises clear images unaffected by noise, while the other part simulates images affected by factors such as stray light and vignetting, which introduce non-uniformities. Given that the algorithm requires a dataset with diverse target representations in various shapes, and real-world data is often limited in diversity due to specific parameters of optical equipment, we will build upon the generation of noise-free base images. Based on the research findings of the Kang-Weiss model [40] and the methodology proposed by Guo et al. [30], we will introduce, through simulation, the non-uniform background effects caused by complex factors, thereby achieving space target images with rich and varied characteristics. This approach not only meets the algorithm’s demand for data diversity but also reflects, to some extent, the complexities found in real-world scenarios.

We first use a normal distribution to represent a clean star map *I*, where the mean μ defines the central point of the brightness values, and the standard deviation σ determines the width of the brightness distribution. Specifically, a larger σ results in a wider spread of brightness values, whereas a smaller σ makes the brightness values more concentrated around the center. By adjusting the parameters of the normal distribution, we can simulate the appearance of the star map under various real-world conditions. Additionally, to better replicate the halo effect of bright stars in the image, we introduce a certain level of Gaussian noise around these stars. This approach not only enhances the realism of the images but also provides more challenging test cases for algorithms.The equation is as follows:

(5) $$\begin{array}{c} f\left(x|\mu ,\sigma \right)=\frac{1}{\sigma \sqrt{2\pi }}e^{-\frac{{\left(x-\mu \right)}^{2}}{2\sigma^{2}}} \end{array}$$

We apply the Kang-Weiss model to simulate the vignetting effect in the images. The formula is as follows:

(6) $$\begin{array}{c} V=A\cdot G\cdot T \end{array}$$

*A* represents the off-axis illumination attenuation law, *G* represents the vignetting effect, and *T* represents the camera tilt. Their respective formulas are as follows:

(7) $$\begin{array}{c} A=\frac{1}{{\left(1+{\left(r/f\right)}^{2}\right)}^{2}},G=1-\alpha r \end{array}$$

(8) $$\begin{array}{c} T=\cos\tau {\left(1+\frac{\tan\tau }{f}\left(u\sin\left(x\right)-v\cos\left(x\right)\right)\right)}^{3} \end{array}$$

In expression *A*, the distance to the center $r={\left(u^{2}+v^{2}\right)}^{1/2}$ is used to describe the distance of a point from the image center, where *u* and *v* represent the horizontal and vertical displacements of the point relative to the center, respectively. By incorporating the effective focal length *f*, vignetting parameter α, and camera tilt angle τ, the physical characteristics of the imaging process can be simulated more accurately. For simulating stray light *S*, the approach proposed by Guo et al. [30] is adopted, which constructs a combined model using Zernike polynomials and the Sigmoid function to effectively characterize random fluctuations on a two-dimensional surface. This method provides a more realistic analysis of the imaging effects, taking into account various non-ideal factors present in optical systems. We have listed the formulas for the Zernike polynomials defined in the Cartesian coordinate system, with the first 10 terms shown below:

(9) $$\begin{array}{cc} z1 & =1\\ z2 & =v\\ z3 & =u\\ z4 & =2uv\\ z5 & =2(u^{2}+v^{2})-1\\ z6 & =-2(u^{2}-v^{2})\\ z7 & =(3u^{2}-v^{2})v\\ z8 & =3u(u^{2}+v^{2})-2u\\ z9 & =3v(u^{2}+v^{2})-2v\\ z10 & =(u^{2}-3v^{2})u\\ \vdots \end{array}$$

Zernike polynomials are typically used for wavefront correction in optical aberrations, where the wavefront shape can be decomposed into a series of orthogonal, axisymmetric terms through Zernike coefficients. Each term represents a different type of aberration, such as a spherical aberration, an astigmatism, a coma, and defocus. However, in this paper, we employ a combination of Zernike polynomials and the Sigmoid function to simulate imaging results under various optical parameters and stray light conditions. The specific formula is provided below:

(10) $$\begin{array}{c} S={\left[\mu \sum_{j=1}^{n}\lambda_{j}Z_{j}\left(u,v\right)+\frac{1}{1+\exp\left(-\sum_{j=1}^{n}\lambda_{j}Z_{j}\left(u,v\right)\right)}\right]}_{\mathrm{nor}} \end{array}$$

where μ serves as a weighting parameter to adjust the intensity of the simulation effect.

Integrating the vignetting model and stray light simulation, our simulation process can be expressed by the following formula: φ=η·V·S+n.

## 3. Experiments and Results

Our experiments were conducted on a device equipped with an Nvidia 24G 4090 GPU. The CPU utilized was an Intel(R) Xeon(R) Platinum 8352V with 16 vCPUs, and the system had 90 GB of RAM. The network architecture was implemented using PyTorch 1.10.0 and Python 3.8.10.

In our training process, we maintain a fixed learning rate of 0.0002 for the first 100 epochs, followed by a linear decay to 0 over the subsequent 100 epochs. For image processing, we initially resize the images to a resolution of 768 × 768 and then randomly crop them to 512 × 512 for input into the network model. Given the characteristics of the CycleGAN framework, we set the batch size to 1; this can be adjusted to match the number of actual GPUs used when multiple GPUs are available. Our experiments indicate that for the CycleGAN framework, a smaller batch size facilitates training and convergence, likely due to the effects of mini-batch stochastic gradient descent. In all testing phases, our image dataset adopts 1024 × 1024 resolution grayscale images for testing.

### 3.1. Evaluation Metrics

#### 3.1.1. PSNR

The Peak Signal-to-Noise Ratio (PSNR) is a commonly used objective metric for evaluating image quality, primarily used to measure the noise level and overall quality of an image. It is often employed to assess the performance of image processing algorithms. The PSNR is defined and calculated based on the Mean Squared Error (MSE). The formula for the PSNR is as follows:

(11) $$\begin{array}{c} \mathrm{PSNR}=10\log_{10}\left(\frac{{\mathrm{MAX}}_{I}^{2}}{\mathrm{MSE}}\right) \end{array}$$

${\mathrm{MAX}}_{I}$ is the maximum possible pixel value of the image. For this experiment, we use 8-bit grayscale images, so ${\mathrm{MAX}}_{I}=255$. The formula for calculating the Mean Squared Error (MSE) is as follows:

(12) $$\begin{array}{c} \mathrm{MSE}=\frac{1}{m\times n}\sum_{i=0}^{m-1}\sum_{j=0}^{n-1}{\left(I(i,j)-K(i,j)\right)}^{2} \end{array}$$

Here, *m* and *n* are the height and width of the image, respectively. I(i,j) represents the pixel value of the original image at position (*i*, *j*), and K(i,j) represents the pixel value of the corrected image at position (*i*, *j*). The PSNR effectively reflects the differences in pixel values at the same positions between the original and corrected images.

#### 3.1.2. SSIM

Unlike the PSNR, which only considers the differences between pixel values, the Structural Similarity Index (SSIM) focuses on the sensitivity of the human visual system to luminance, contrast, and structural information. It places more emphasis on the structural information of the image, thus often providing a better reflection of the subjective quality of the image. The formula for calculating the SSIM is as follows:

(13) $$\begin{array}{c} \mathrm{SSIM}\left(x,y\right)=\frac{\left(2\mu_{x}\mu_{y}+C_{1}\right)\left(2\sigma_{xy}+C_{2}\right)}{\left(\mu_{x}^{2}+\mu_{y}^{2}+C_{1}\right)\left(\sigma_{x}^{2}+\sigma_{y}^{2}+C_{2}\right)} \end{array}$$

Here, *x* and *y* are the original image and the processed image, respectively. $\mu_{x}$ and $\mu_{y}$ are the means of *x* and *y*, respectively. $\sigma_{x}^{2}$ and $\sigma_{y}^{2}$ are the variances of the two images, and $\sigma_{xy}$ is the covariance of *x* and *y*. $C_{1}$ and $C_{2}$ are constants used to stabilize the denominator and prevent division by zero errors.

#### 3.1.3. RMSE and MAE

In the field of image processing, the Root Mean Square Error is a measure that calculates the square root of the mean of the squared differences between pixel values of the reference image and the actual image. It tends to be more sensitive to larger error values. The formula for the RMSE is as follows:

(14) $$\begin{array}{c} \mathrm{RMSE}=\sqrt{\frac{1}{N}\sum_{i=1}^{N}{\left(y_{i}-{\hat{y}}_{i}\right)}^{2}} \end{array}$$

The Mean Absolute Error represents the average of the absolute differences between the pixel values of the reference image and the true image. Compared to the RMSE, the MAE is less sensitive to large errors but provides an overall assessment of the difference between two images. The formula for the MAE is given below:

(15) $$\begin{array}{c} \mathrm{MAE}=\frac{1}{N}\sum_{i=1}^{N}\left|y_{i}-{\hat{y}}_{i}\right| \end{array}$$

Here, $y_{i}$ denotes the reference image, ${\hat{y}}_{i}$ represents the actual image, and *N* stands for the number of samples.

#### 3.1.4. FID

FID (Fréchet Inception Distance) is a widely recognized evaluation metric primarily used to assess the quality of images generated by Generative Adversarial Networks (GANs). Its popularity stems from the strong correlation between FID scores and human subjective evaluations of image quality. By calculating the distance between the feature distributions of the generated image dataset and the real image dataset, FID quantifies the discrepancy between their multivariate Gaussian distributions. Specifically, FID employs a formula to measure the difference between the data distribution generated by the model and the target real data distribution. The formula is as follows:

(16) $$\begin{array}{c} \mathrm{FID}\left(\mu_{r},\Sigma_{r},\mu_{g},\Sigma_{g}\right)={∥\mu_{r}-\mu_{g}∥}_{2}^{2}+\mathrm{Tr}(\Sigma_{r}+\Sigma_{g}-2{\left(\Sigma_{r}\Sigma_{g}\right)}^{\frac{1}{2}}) \end{array}$$

where $\left(\mu_{r},\Sigma_{r}\right)$ and $\left(\mu_{g},\Sigma_{g}\right)$ denote the sample mean and the covariance matrix of the features approximating the real data and generated data, respectively. Tr represents the trace operation of a matrix [41]. FID utilizes a pre-trained Inception V3 network to extract image features. Specifically, instead of using the final layer’s classification output for computation, it adopts the activations from the second-to-last pooling layer as the feature representation of the images. The smaller the FID is, the closer the features are, indicating that the transformed images are more realistic.

#### 3.1.5. The Metrics on the Real Image

Evaluating the quality of real images in the absence of a reference image for comparison can be quite challenging. To tackle this issue, we have implemented an approach that utilizes an adaptive threshold segmentation algorithm. This method processes images after various techniques have been used to correct their non-uniform backgrounds, aiming to isolate a pure noise background. By subsequently calculating the mean and standard deviation of this noise background, we can assess the effectiveness of the different correction methods. Generally speaking, lower mean and standard deviation values indicate superior correction performance. Specifically, the formula for calculating the threshold *T* is as follows:

(17) $$\begin{array}{c} T=\mu +\alpha \sigma \end{array}$$

where α is a constant coefficient, μ and σ are the mean and standard deviation of the noise background image, respectively. After obtaining the threshold *T*, we can perform threshold segmentation on the image. The formula for this process is as follows:

(18) $$\begin{array}{c} b\left(i,j\right)=\left\{\begin{array}{cc} 0, & f\left(i,j\right)<T_{i}\\ 1, & f\left(i,j\right)\geq T_{i} \end{array}\right. \end{array}$$

Here, f(i,j) represents the image to be processed, and b(i,j) is the binary image of f(i,j). We convert this binary image into a Boolean mask. Using this mask, we filter the original image, retaining only the pixels that satisfy the condition; other positions are filled with zero-valued pixels. The resulting image is the segmented image. The formula is as follows:

(19) $$\begin{array}{c} f^{\prime }\left(i,j\right)=\left\{\begin{array}{cc} f(i,j), & f\left(i,j\right)\geq T_{i},\\ 0, & f\left(i,j\right)<T_{i}. \end{array}\right. \end{array}$$

$F^{\prime }\left(i,j\right)$ represents the image obtained after threshold segmentation, which contains a noisy background. We calculate the mean $\mu_{\mathrm{seg}}$ and standard deviation $\sigma_{\mathrm{seg}}$ of this image to reflect the correction effect of different algorithms on the true image.

### 3.2. Ablation Experiments

In this section, we will conduct ablation studies to validate the effectiveness of our algorithm. Our network framework is based on the CycleGAN, which serves as our baseline model. Compared to the original CycleGAN, we have made improvements in the initial layers and the backbone network (ResNet). We will sequentially evaluate the impact of these modifications on model performance.

In the generator design of the CycleGAN, an initial convolutional layer with a 7 × 7 kernel is employed to provide a large receptive field, effectively capturing broad spatial information. However, for spatial target images, our focus lies on detecting small targets distributed as points or lines, which are not only minute in size but also have pixel intensities that can be comparable to noise levels. Therefore, it is critical to enhance attention to finer-grained features using smaller convolutional kernels to better extract local characteristics. Additionally, given the non-uniformity of backgrounds in images, which varies due to different parameters and environmental factors, the network must also capture spatial dependencies across multiple scales.

To address these requirements, we introduce a multi-scale feature extraction module (MSFEM) designed to optimize feature extraction at various scales. To validate its effectiveness, ablation studies were conducted. As shown in Table 1, the experimental results demonstrate that our proposed network architecture outperforms alternative methods. Specifically, when the ratio of 3 × 3, 5 × 5, and 7 × 7 kernels is set to 2:1:1, the network efficiently integrates both fine-grained and coarse-grained features, achieving optimal performance.

In summary, by strategically configuring kernels of different sizes, our network not only captures global spatial information effectively but also significantly enhances sensitivity to small targets and background variations, leading to more precise and detailed feature extraction.

Given the high resolution of our network’s input images, coupled with the relative scarcity of useful information, the sparse distribution of target stars, minute shapes, and simple contours, along with non-uniform backgrounds that vary in form and continuity, the initial stage of information extraction becomes particularly critical. This phase must balance both short-range and long-range spatial dependencies to effectively capture the sparse and minute target features. To address these challenges, we have designed a multi-scale feature extraction module (MSFEM) that is specifically optimized for feature extraction at this critical stage.

To enhance the processing of high-dimensional feature maps, we have introduced efficient multi-scale attention (EMA). This mechanism reallocates feature weights, enabling the network to learn across space and focus on the most critical areas. The EMA significantly enhances the network’s feature extraction capabilities without substantially increasing computational load or model complexity. This makes it ideal for integration into the backbone network and makes it well-suited to meet our task requirements.

To validate the effectiveness of our proposed method, we incrementally incorporated the MSFEM and the EMA-residual block into the baseline model. As shown in Table 2, where EMA_8 denotes eight groups, the results indicate that although the PSNR values fluctuate slightly with the increase in the number of groups, other metrics show significant improvements. Our analysis suggests that as the number of groups increases, the number of channels per group decreases, allowing each subgroup to focus better on learning fine-grained local features. Moreover, through independent group normalization and convolution operations, this implies the presence of more independent nonlinear paths, enhancing the overall model’s nonlinear expression capability and leading to more accurate predictions. Considering the final results, our approach, which combines the MSFEM and EMA_32, excels across all performance metrics: achieving a peak PSNR value of 32.7923, an SSIM score of 0.9814, and the lowest FID score of 1.9212.

In summary, by integrating the MSFEM and the EMA mechanism, our network model can efficiently capture sparse small-target features in high-resolution images while maintaining low computational costs. This combination provides a precise and effective solution for feature extraction, showcasing an optimal balance between performance and efficiency.

### 3.3. Results on Simulation Data

We selected the classic Top-Hat algorithm and several state-of-the-art unpaired image translation deep learning algorithms—CycleGAN, U-GAT-IT, and StegoGAN—for comparison. Using their open-source code, we conducted model training and testing. Table 3 presents the quantitative evaluation metrics of each algorithm on the test set, demonstrating that our proposed model outperforms all others across the board.

Specifically, our model achieved PSNR, SSIM, RMSE, and MAE scores of 32.7923, 0.9814, 5.9154, and 17.5372, respectively, significantly surpassing other methods. Compared to the best-performing CycleGAN, our model’s PSNR was 8.3952 higher, and its SSIM score was 0.2089 higher. Moreover, our model achieved the lowest RMSE and MAE values. In terms of the FID metric, among the four deep learning algorithms, our model attained the lowest score of 1.9212, further highlighting its superiority. And our approach, when compared on metrics such as FLOPs, inference time, and model parameters, is slightly inferior to the CycleGAN model but significantly outperforms other deep learning models.

In summary, our algorithm performs excellently in the aforementioned metrics, demonstrating that our approach is highly effective for the task of processing spatial target image non-uniformity. In summary, the experimental results confirm that our algorithm excels in all evaluation metrics, showcasing its outstanding performance.

**Figure 4:**
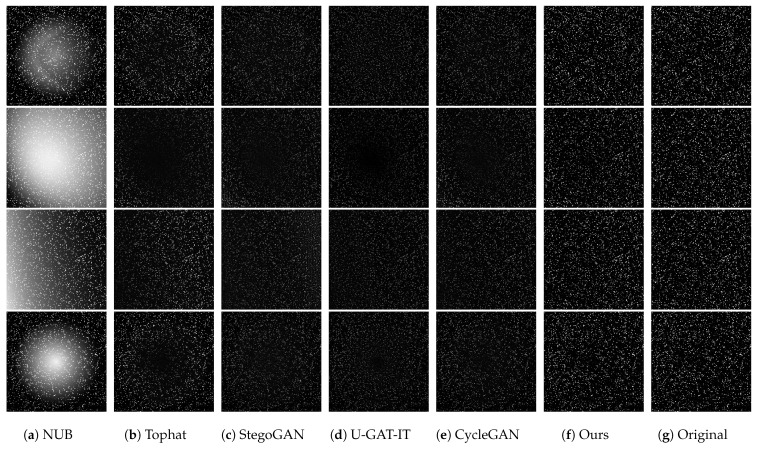
Correction results of various methods on simulated images with differing non-uniform background (NUB) intensities. (**a**) is the image with a simulated non-uniform background, (**b**–**f**) are images processed by different methods, and (**g**) is the original uniform image.

In this study, we provide four simulated space object images that feature varying degrees of non-uniform background intensity. These images, along with their corresponding original images, are used for a subjective evaluation of different models’ ability to correct non-uniform backgrounds. As illustrated in Figure 4, we compare the effects of several correction methods applied to these four simulated images concerning non-uniform background correction. The performance differences among various methods can be intuitively observed from the correction results.

When the non-uniform effect in the image is relatively mild (as seen in the first image), all five methods achieve effective correction to some extent. However, when dealing with severe non-uniform effects (such as those shown in the second image), it becomes apparent that several methods, including top-hat, mistakenly eliminate certain small targets while reducing the central contrast of background brightness. These small targets partially merge into the background due to the influence of non-uniformity. StegoGAN and U-GAT-IT face similar issues, though CycleGAN performs relatively better. Still, it struggles with correcting backgrounds that have multi-scale variations within the image. In stark contrast, our method effectively corrects non-uniform backgrounds even under extreme conditions, while preserving the integrity of faint targets with low signal-to-noise ratios and ensuring that the corrected image exhibits a uniform distribution overall.

For the third and fourth images, Tophat, StegoGAN, and U-GAT-IT fail to ensure overall uniformity when handling edge or locally gradient non-uniform effects; the original CycleGAN barely achieves this but at the cost of overall image brightness and contrast, which is not the desired outcome. Results processed by our method show that it can maintain an overall brightness and contrast that are close to the original levels while performing corrections, ensuring clear contours of space objects and well-preserved faint targets. This is crucial for the subsequent detection of faint targets, as it not only retains the target’s effective information and outline but also enhances the target’s signal-to-noise ratio.

To sum up, our method excels in correcting non-uniform effects across a variety of image conditions. It successfully retains the integrity of the targets and maintains the overall uniformity of the image, on par with the original images. To substantiate this claim, we performed a difference operation between the corrected images and the originals to isolate the non-uniform background components. We then compared these results against the true non-uniform background obtained from our simulations.

**Figure 5:**
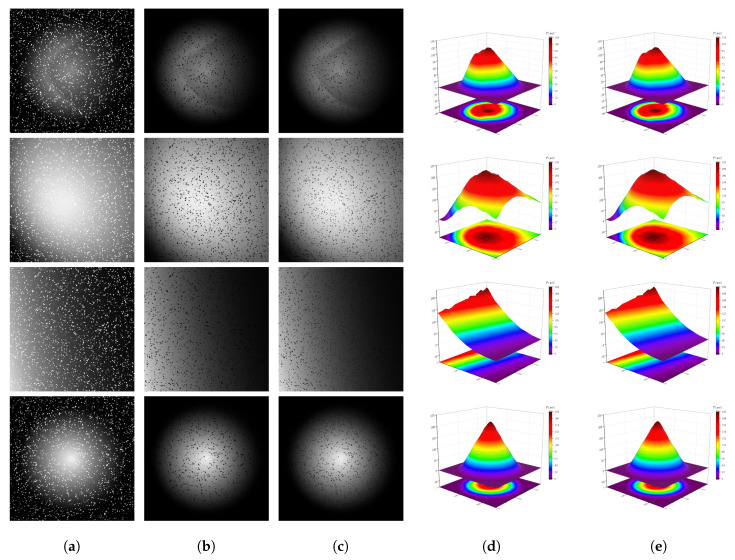
Comparison of non-uniform backgrounds and their heatmaps. (**a**) represents generated nonuniform images. (**b**) A ground truth image of the non-uniform background. (**c**) The background image inferred by our method. (**d**,**e**) are 3D heatmaps of the ground truth image of the non-uniform background and the background image inferred by our method.

As shown in Figure 5, we compared the correction effect of our method with the true background conditions. The contents displayed include a synthetically generated non-uniform image, the non-uniform background truth of this image, and the inferred non-uniform background image obtained through our method. From the comparison, it can be observed that the background noise extracted after correction by our method is very close to the theoretically expected noise background. To more intuitively emphasize this point, we used three-dimensional heat maps for further demonstration: Figure 5d shows the background truth, while Figure 5e presents the background image inferred by our method. It can be clearly seen through observation that the inferred background and the actual non-uniform background energy distribution are almost completely consistent, i.e., the majority of the grayscale values at different positions are either the same or only slightly different. This indicates that our method has very good effectiveness and accuracy in handling non-uniform background correction.

### 3.4. Results on Real Data

**Figure 6:**
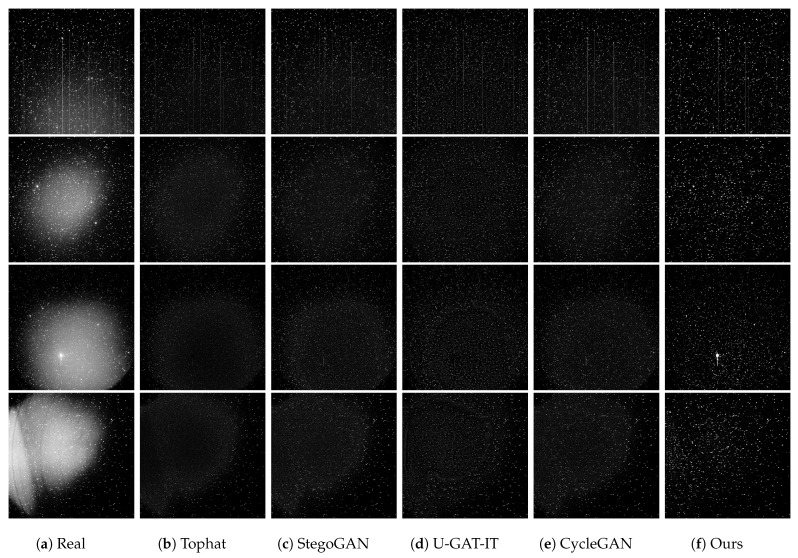
Results of different methods on real images. (**a**) is the real image captured by a ground-based telescope. (**b**–**f**) are the results of correction using different methods.

To evaluate the practical effectiveness of our proposed method, we selected four representative space images as test samples. These images contain non-uniform backgrounds of varying intensities and shapes to ensure a diverse and representative set of experimental conditions. We applied four existing methods alongside our proposed method to perform non-uniform background correction on these real datasets, with the comparative results presented in Figure 6.

When the impact of the non-uniform background is mild, all five methods effectively preserve target information, maintaining clear target contours and high edge contrast while successfully correcting the non-uniform background. However, as the intensity of the non-uniform background increases, the performance of each method begins to diverge. The Tophat transformation tends to reduce overall image brightness and contrast when handling complex backgrounds, leading to the erroneous elimination of faint targets, which is highly detrimental for subsequent target detection. The StegoGAN exhibits similar issues. In contrast, although U-GAT-IT and the CycleGAN mitigate this problem to some extent, they leave noticeable correction artifacts and residual non-uniform background contours in the corrected images, introducing many noise pixels that increase false alarms in later stages of target detection and tracking. Our method, however, can handle non-uniform backgrounds of any energy level and shape while fully preserving the clear contours and intensity features of the targets, leaving virtually no correction artifacts. This approach not only improves the signal-to-noise ratio (SNR) of the targets but also provides higher quality data support for follow-up analyses.

For the quality assessment of images without a reference, choosing an appropriate method is critical, as an inappropriate choice can lead to the misjudgment of the final results. To address this, we adopted a method for extracting residual noise from the background by using adaptive threshold segmentation to remove most of the target pixels from the corrected image, thereby obtaining the noise background. We then calculated the mean and standard deviation of this noise background. Table 4 shows that our algorithm performs best in both metrics, with a mean value of 1.3482 and a standard deviation of 4.9725, indicating superior algorithmic performance.

**Figure 7:**
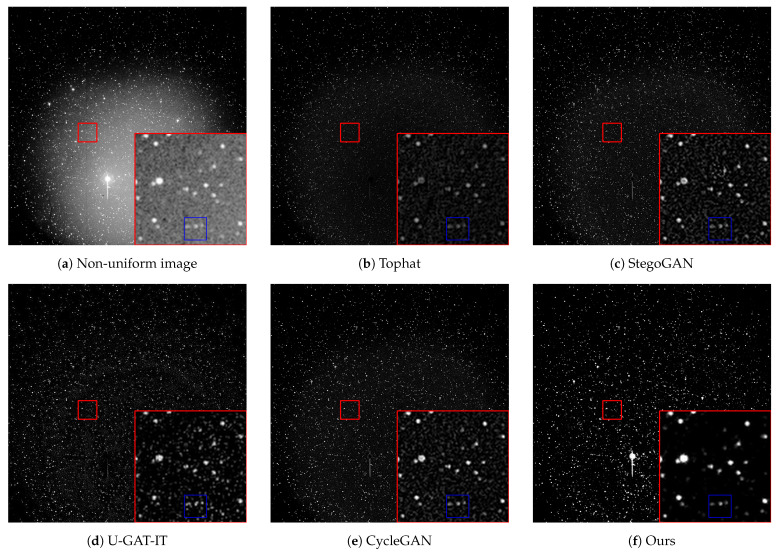
Correction of local details in non-uniform images with various methods. The red area is a magnified section of the original image, and the blue area is a marking within the red area that contains three targets.

To evaluate the performance of our algorithm in correcting local non-uniformities in images, we selected a region from the third image and compared it after a 10-fold magnification, as shown in Figure 7. The selected area was marked with a red square, magnified, and placed at the lower right corner for comparison. Results demonstrate that our algorithm is significantly more effective in suppressing local non-uniform background noise than other methods. Specifically, in the detail marked by the blue box, three dim targets within an image containing non-uniform background noise are nearly obscured by the noise. While other algorithms offer some improvement, they also leave varying degrees of correction artifacts, which can interfere with subsequent detection tasks. In contrast, our method not only preserves the targets intact but also minimizes correction artifacts.

In summary, our method demonstrates outstanding performance in addressing non-uniform background issues in real images, providing a solid foundation for improving the accuracy of space target detection and recognition.

## 4. Discussion

In our previous discussions, we explored the application of conditional Generative Adversarial Networks (cGANs) such as pix2pix, referencing the work by Guo et al. [30]. At that time, we opted for a supervised learning approach, utilizing extensive datasets with manually annotated pairs to train the network. By incorporating cascading methods and skip connections, we aimed to enhance network performance and mitigate overfitting risks. However, this method has limitations due to its heavy reliance on dataset quality; suboptimal datasets can significantly impact model performance on real images.

To address these challenges, our latest research pivoted towards unsupervised learning, focusing on correcting non-uniform backgrounds in visible-light space object images captured by ground-based telescopes. Despite the relatively small dataset—comprising only 1000 synthetic images per image domain for training—the results have been remarkably positive. This success is attributed to our focused investigation into key factors affecting correction outcomes: scale issues. Non-uniform backgrounds are typically caused by varying shooting conditions, optical equipment parameters, and random stray light, all of which manifest across multiple scales within images. Therefore, our research concentrated on optimizing correction effects at multiple scales while maintaining relatively low computational resources and parameter counts.

Specifically, we introduced three different sizes of convolution kernels in the initial convolutional layers of the network to extract image feature information at multiple scales from the very first layer, followed by pointwise convolutions for channel information fusion and dimensionality reduction. Furthermore, an efficient multi-scale attention (EMA) mechanism was embedded within the ResNet backbone, enabling the network to perform multi-scale information extraction and cross-spatial learning in high-dimensional feature maps without significantly increasing parameters or computational costs. The experimental results demonstrated the effectiveness of this strategy, with it proving to be successful even without a large annotated dataset. Our model is relatively easy to generalize to other fields with noise variations and low target energy. As for the non-uniform effects under the microscope mentioned by Smith et al. [42], the loss function and parameters need to be adjusted to ensure the invariance of the color domain and edge texture during the image domain transfer process.

## 5. Conclusions

Looking ahead, our research will explore Vision Transformer (ViT) and diffusion models. Given the limited computational resources and the goal of achieving superior results with smaller datasets and less computational effort, we have not yet delved deeply into the performance of Transformer architectures and diffusion models, as they also require substantial data and computational resources. Our objective is to ensure that the main edges of space objects remain clear and that the signal-to-noise ratio improves while using ViTs and diffusion models to effectively correct non-uniform backgrounds. This represents the primary direction for our future research endeavors. Additionally, in the selection of evaluation metrics, we have drawn inspiration from the metric fusion approach proposed by Stanciu et al. [43]. One of our future research directions will involve developing a fusion metric that is suitable for space object images, to serve as an effective standard for assessing correction outcomes, thereby enabling a more accurate evaluation of image correction quality. 

## Figures and Tables

**Table 1:** Impact of different kernel combinations on MSFEM network performance.

Name	Kernel Size = 3	Kernel Size = 5	Kernel Size = 7	PSNR	SSIM	RMSE	MAE	FID
MSFEM-1	√			25.6282	0.8862	13.3634	49.9480	10.9881
MSFEM-2		√		24.1676	0.8610	15.8222	54.5638	16.6394
MSFEM-3			√	24.3971	0.7725	15.3997	64.3716	21.5845
MSFEM-4	√	√		24.4509	0.8458	15.3120	68.8082	14.1705
MSFEM-5		√	√	17.0526	0.6383	35.8191	50.2485	39.2884
MSFEM-6	√		√	24.5707	0.8702	15.0835	43.6171	15.7936
MSFEM	√	√	√	31.1229	0.9792	7.1463	17.0587	2.1292

Note: The symbol √ indicates that the convolution kernel is included.

**Table 2:** Ablation study performance comparison of baseline model with incremental addition of MSFEM and EMA-residual block.

Name	PSNR	SSIM	RMSE	MAE	FID
Baseline	24.3971	0.7725	15.3997	64.3716	21.5845
Baseline + MSFEM	31.1229	0.9792	7.1463	17.0587	2.1292
Baseline + EMA_8	28.2701	0.9426	9.9108	24.3276	5.5416
Baseline + EMA_16	28.2642	0.9437	9.9163	24.3824	5.5829
Baseline + EMA_32	28.2503	0.9613	9.9294	24.3123	5.4217
Ours (full)	32.7923	0.9814	5.9154	17.5372	1.9212

**Table 3:** Comparative analysis of metric results across different methods.

Method	PSNR	SSIM	RMSE	MAE	FID	FLOPs (G)	Inference Time (ms)	Parameters (M)
TopHat	14.3405	0.6113	48.9275	122.8254	N/A	N/A	0.98	N/A
CycleGAN	24.3971	0.7725	15.3997	64.3716	21.5845	332	33.88	7.82
UGATIT	19.1275	0.6454	28.2006	18.6646	120.5023	569	90.37	15.29
StegoGAN	19.9319	0.6555	25.7622	109.4055	101.0250	448	53.61	11.37
Ours	32.7923	0.9814	5.9154	17.5372	1.9212	341	51.39	7.84

**Table 4:** Correction results of different methods on real non-uniform images.

	Original	Tophat	StegoGAN	U-GAT-IT	CycleGAN	Ours
$\mu_{\mathrm{seg}}$	51.9086	5.8793	5.5892	3.7581	5.1887	1.3482
$\sigma_{\mathrm{seg}}$	56.8238	8.2345	9.5476	7.4898	8.9484	4.9725

## Data Availability

The data presented in this study are available on request from the corresponding author due to privacy.
